# Empty Reviews: A Description and Consideration of Cochrane Systematic Reviews with No Included Studies

**DOI:** 10.1371/journal.pone.0036626

**Published:** 2012-05-04

**Authors:** Joanne Yaffe, Paul Montgomery, Sally Hopewell, Lindsay Dianne Shepard

**Affiliations:** 1 College of Social Work, University of Utah, Salt Lake City, Utah, United States of America; 2 Centre for Evidence-Based Intervention, University of Oxford, Oxford, United Kingdom; 3 Centre for Statistics in Medicine, University of Oxford, Oxford, United Kingdom; University of British Columbia, Canada

## Abstract

**Background:**

There is no specific guidance for the reporting of Cochrane systematic reviews that do not have studies eligible for inclusion. As a result, the reporting of these so-called “empty reviews” may vary across reviews. This research explores the incidence of empty systematic reviews in *The Cochrane Database of Systematic Reviews (The CDSR)* and describes their current characteristics.

**Methodology/Principal Findings:**

Empty reviews within *The CDSR* as of 15 August 2010 were identified, extracted, and coded for analysis. Review group, original publication year, and time since last update, as well as number of studies listed as excluded, awaiting assessment, or on-going within empty reviews were examined. 376 (8.7%) active reviews in *The CDSR* reported no included studies. At the time of data collection, 45 (84.9%) of the Cochrane Collaboration's 53 Review Groups sustained at least one empty review, with the number of empty reviews for each of these 45 groups ranging from 1 to 35 (2.2–26.9%). Time since original publication of empty reviews ranged from 0 to 15 years with a mean of 4.2 years (SD = 3.4). Time since last assessed as up-to-date ranged from 0 to 12 years with a mean of 2.8 years (SD = 2.2). The number of excluded studies reported in these reviews ranged from 0 to 124, with an average of 9.6 per review (SD = 14.5). Eighty-eight (23.4%) empty reviews reported *no* excluded studies, studies awaiting assessment, or on-going studies.

**Conclusions:**

There is a substantial number of empty reviews in *The CDSR*, and there is some variation in the reporting and updating of empty reviews across Cochrane Review Groups. This variation warrants further analysis, and may indicate a need to develop guidance for the reporting of empty systematic reviews in *The CDSR*.

## Introduction

The Cochrane Library is the largest and perhaps best recognized global collection of health care evidence, currently hosting more than 4,500 systematic reviews in its *Cochrane Database of Systematic Reviews (CDSR)*. However, it has been reported that clinicians find Cochrane reviews of limited relevance to practice decisions. For example, one study found that while Cochrane reviews are highly regarded for their quality, they are used less than other sources for clinical decision-making because of their emphasis on methodology and rigor rather than on clinical relevance [1].

It is not Cochrane's policy to provide guidelines for practice or policy decisions [2]. Instead, it sees itself as the provider of best quality evidence and specifically states that guidelines go “beyond a systematic review and require additional information and informed judgments that are typically the domain of clinical practice guideline developers.”

Systematic reviews that find no studies eligible for inclusion, commonly known as “empty reviews,” may be especially problematic for clinicians and other decision-makers. Little is known about the incidence, prevalence or variation in reporting of such reviews [3]. The little that has been written about them suggests that the reporting of implications for practice may sustain a risk for bias. With no studies meeting criteria for inclusion, these empty reviews may appear to: (1) offer no conclusions, (2) offer conclusions based on referenced excluded studies, (3) offer conclusions based on other evidence, or (4) offer conclusions not based on evidence. Thus, empty reviews may contribute to what appears to be generalized disappointment with *The CDSR* among some clinicians and policymakers [1], [4].

### Issues Related to Empty Reviews

In examining the literature concerning empty reviews, we offer the following summary of the core issues. First, empty reviews may relate to an area of study which is very new. Cooper asserts that research syntheses should concern topics for which there is already a body of evidence [4]. Where it is important to identify new interventions and gaps in knowledge, systematic reviews provide direction for targeted research and in some countries, may be required as part of large grant applications for trials.

Second, in some circumstances, reviews may focus on questions that are highly specific. For example, they may restrict the population by age, context, diagnostic criteria, or intervention criteria. In the case that studies meeting these specified criteria have not been conducted, there are no includable studies.

Third, many empty reviews may be the result of overly stringent methodological inclusion criteria imposed in the interest of higher quality evidence. These criteria may involve study selection based on specific designs, outcome measures, or comparison conditions which may not be available in existing primary studies.

The issue of empty reviews was introduced to the literature in 2007 by Lang and colleagues, who suggested that guidelines were needed for reporting of empty reviews in order to prevent reviewers from deriving unsubstantiated implications for practice, or from simply concluding that no eligible studies were found [3]. Lang et al. further suggest that in the case of empty reviews, authors should note observations from ineligible articles and abstracts. In response, Green et al., while acknowledging that “a specific structure for the reporting of empty reviews and providing information for further research could be helpful,” argue that basing conclusions on studies which do not meet inclusion criteria specified in the review protocol increases the risk of bias of the review and may, indeed, mislead readers [5]. In an editorial supporting the inclusion of empty systematic reviews in *Evidence-based Communication Assessment and Intervention*, Schlosser and Sigafoos concur with Green and colleagues' position, and encourage commentators for the journal “to highlight this potential for biases if an empty review over-reached in their analysis and interpretation of excluded studies.” [6]

The *Cochrane Handbook for Systematic Reviews of Interventions* sets policy and provides specific guidance for the reporting of Cochrane systematic reviews but does not yet provide specific guidance for the reporting of empty reviews [2]. As a result, the reporting of empty reviews may be inconsistent.

### Study Aims

The objectives of the present study were to provide a description of empty reviews and their general characteristics in *The CDSR* and across topic areas as defined by Cochrane Review Groups. To explore the extent to which empty reviews are reported in *The CDSR*, we first identified all reviews without included studies and examined their frequency and proportion overall as well as within Cochrane Review Groups. Second, to examine the persistence of empty reviews, we analyzed time since original publication of identified reviews. Third, to assess the level of existing, but non-includable, research related to topics of empty reviews, we examined the number of excluded studies reported by each of these reviews across *The CDSR* and within Cochrane Review Groups. Finally, to assess the possibility of future updating with eligible studies, we examined time since last update of these reviews as well as numbers of reported on-going studies and studies awaiting assessment. In this way, we hoped to establish whether the prevalence and general characteristics of empty reviews varied systematically across Cochrane Review Groups. We assumed that inconsistencies in prevalence and characteristics across Cochrane Review Groups might suggest the necessity of general guidelines for the reporting of empty reviews in *The CDSR*.

## Methods

The Cochrane Collaboration Information Management System (Archie) was used to identify any Cochrane systematic reviews through December 2009 that contained no included studies. These reviews were verified as empty by two authors. Remaining reviews from January to August 15, 2010 were identified through hand search of *The CDSR* by one author and verified by a second author. PDF versions of empty reviews were downloaded and data extracted from relevant sections of reviews by one author and verified by a second author. Age of reviews was calculated in years between the original publication year and 2010. Time since last update was calculated in years between the date reported in the history section of each review and August 15, 2010. Data calculations were performed by one author and verified by a second author. Any disagreements between the two authors were resolved by discussion.

Data were entered into Excel spreadsheets and exported to PASW Statistics, version 16 (IBM, Somers, NY) to provide descriptive statistics. Differences across Cochrane Review Groups were detected by visual analysis.

## Results

*The CDSR* contained a total of 4,320 systematic reviews on August 15, 2010, of which 376 (8.7%) reported no studies eligible for inclusion – that is, were empty reviews. Forty-five (84.9%) of the Cochrane Collaboration's 53 Review Groups hosted at least one empty review, with the number of empty reviews within these 45 Review Groups ranging from 1 to 35 (proportionately 2.2% to 26.9%).

Eight Cochrane Review Groups did not host any empty reviews, including the Back, Fertility Regulation, Haematological Cancers, Methodology, Occupational Safety and Health, Prostatic Diseases and Urologic Cancers, Public Health, and Sexually Transmitted Diseases Groups. In contrast, several Cochrane Review Groups sustained higher numbers of these empty reviews, although raw numbers of empty reviews can only be understood in the context of the total numbers of reviews supported by these groups. For example, the Pregnancy and Childbirth Group listed the largest number of empty reviews with 35, but this number represented only 8.9% of their 394 published reviews. Table 1 displays the total number of systematic reviews, number of empty reviews, and percentage of reviews which were empty for each of the 53 Cochrane Review Groups on August 15, 2010. Distribution of empty reviews varied considerably across groups. There were four groups hosting particularly large absolute numbers of empty reviews, including the Pregnancy and Childbirth, Airways, Cystic Fibrosis and Genetic Disorders, and Neonatal Groups, while six groups hosted relatively high percentages of empty reviews, including the Cystic Fibrosis and Genetic Disorders, Childhood Cancer, Eyes and Vision, Developmental and Psychosocial Learning Problems, Consumers and Communication, Neuromuscular Diseases, and Oral Health Groups.

**Table 1:** Reviews and Empty Reviews by Cochrane Review Group on August 15, 2010.

Cochrane Review Group	Total # of Reviews	# (%) of Empty Reviews
Acute Respiratory Infections	109	3 (2.8)
Airways	223	26 (11.7)
Anaesthesia	65	4 (6.2)
Back	52	0 (0.0)
Bone, Joint and Muscle Trauma	92	3 (3.3)
Breast Cancer	38	1 (2.6)
Childhood Cancer	8	2 (25.0)
Colorectal Cancer	67	3 (4.5)
Consumers and Communication	29	5 (17.2)
Cystic Fibrosis and Genetic Disorders	93	25 (26.9)
Dementia and Cognitive Improvement	88	12 (13.6)
Depression, Anxiety and Neurosis	111	6 (5.4)
Developmental, Psychosocial and Learning Problems	81	15 (18.5)
Drugs and Alcohol	53	3 (5.7)
Ear, Nose and Throat Disorders	65	7 (10.8)
Effective Practice and Organisation of Care	68	5 (7.4)
Epilepsy	54	6 (11.1)
Eyes and Vision	80	19 (23.8)
Fertility Regulation	60	0 (0.0)
Gynaecological Cancer	85	7 (8.2)
Haematological Malignancies	21	0 (0.0)
Heart	87	4 (4.6)
Hepato-Biliary	117	10 (8.5)
HIV/AIDS	67	6 (9.0)
Hypertension	37	2 (5.4)
Incontinence	66	2 (3.0)
Infectious Diseases	99	4 (4.0)
Inflammatory Bowel Disease and Functional Bowel Disorders	55	2 (3.6)
Injuries	103	14 (13.6)
Lung Cancer	25	3 (12.0)
Menstrual Disorders and Subfertility	150	8 (5.3)
Metabolic and Endocrine Disorders	72	5 (6.9)
Methodology Review	14	0 (0.0)
Movement Disorders	49	6 (12.2)
Multiple Sclerosis	28	2 (7.1)
Musculoskeletal	137	3 (2.2)
Neonatal	260	23 (8.8)
Neuromuscular Disease	83	14 (16.9)
Occupational Safety and Health	0	0 (0.0)
Oral Health	108	18 (16.7)
Pain, Palliative and Supportive Care	134	16 (11.9)
Peripheral Vascular Disease	80	5 (6.3)
Pregnancy and Childbirth	394	35 (8.9)
Prostatic Diseases and Urologic Cancers	31	0 (0.0)
Public Health	1	0 (0.0)
Renal	85	3 (3.5)
Schizophrenia	148	18 (12.2)
Sexually Transmitted Diseases	6	0 (0.0)
Skin	48	3 (6.3)
Stroke	128	7 (5.5)
Tobacco Addiction	53	3 (5.7)
Upper Gastrointestinal and Pancreatic Diseases	47	3 (6.4)
Wounds	66	5 (7.6)
**All Review Groups**	**4320**	**376 (100.0)**

**Figure 1:**
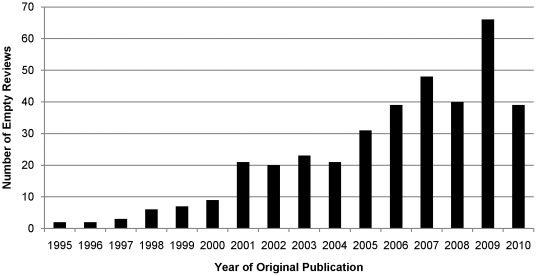
Empty Reviews by Year of Original Publication, as of August 15, 2010 (*N* = 376).

Figure 1 presents the distribution of empty reviews by date of original publication. Time since original publication of empty reviews ranged from 0 to 15 years with a mean of 4.2 years (*SD* = 3.4). Twenty of these reviews have remained empty from the 1990 s and 113 from before 2005. One hundred forty-five (38.6%) reviews were more recent and have been published since 2008. While most empty reviews were less than 5 years old, 28 (7.4%) reviews were 10 years or older.

Table 2 summarizes other characteristics of empty reviews identified by the study. Time since empty reviews were last assessed as up-to-date ranged from 0 to nearly 12 years with a mean of 2.8 years (*SD* = 2.2). One hundred eighty-three (48.7%) reviews had been updated in the last 2 years. One hundred ninety-three (51.3%) reviews had not been updated in more than 2 years; 56 (14.9%) had not been updated in more than 5 years; and 6 (1.6*%)* had not been updated in more than 10 years. There was some variability across Cochrane Review Groups with respect to average time since updating empty reviews. For example, empty reviews for the Cystic Fibrosis and Genetic Disorders Group averaged less than a year since update (*M* = 0.9, *SD* = 0.7), while the average time since updating empty reviews for the Skin Group was more than 7 years (*M* = 7.3; *SD* = 5.4). It should be noted that these statistics are not reflective of Cochrane Review Groups' updating of all reviews, as empty reviews represent only a small proportion of reviews published by these groups.

**Table 2:** Characteristics of Empty Reviews by Cochrane Review Group on August 15, 2010.

Cochrane Review Groups Hosting Empty Reviews	# of Empty Reviews	Time (Years) Since Last Up-Dated		# of Excluded Studies		# of On-going Studies		# of Studies Awaiting Assessment	
		*M*	*(SD)*	*M*	*(SD)*	*M*	*(SD)*	*M*	*(SD)*
Acute Respiratory Infections	3	3.3	(1.8)	32.0	(23.6)	0.0	(0.0)	0.0	(0.0)
Airways	26	1.7	(1.0)	6.9	(5.3)	0.1	(0.3)	0.0	(0.0)
Anaesthesia	4	2.4	(1.5)	4.3	(7.8)	0.0	(0.0)	0.0	(0.0)
Bone, Joint and Muscle Trauma	3	3.4	(2.1)	4.0	(4.4)	0.0	(0.0)	0.0	(0.0)
Breast Cancer	1	3.9	(0.0)	23.0	(0.0)	0.0	(0.0)	0.0	(0.0)
Childhood Cancer	2	1.6	(0.5)	9.0	(5.7)	0.0	(0.0)	0.0	(0.0)
Colorectal Cancer	3	3.6	(2.8)	4.3	(6.7)	0.0	(0.0)	0.0	(0.0)
Consumers and Communication	5	5.2	(2.0)	23.8	(42.4)	0.0	(0.0)	0.0	(0.0)
Cystic Fibrosis and Genetic Disorders	25	0.9	(0.7)	3.9	(7.3)	0.0	(0.0)	0.1	(0.3)
Dementia and Cognitive Improvement	12	2.3	(1.0)	11.9	(17.0)	0.3	(0.7)	0.2	(0.6)
Depression, Anxiety and Neurosis	6	2.7	(0.8)	6.3	(6.1)	0.0	(0.0)	0.0	(0.0)
Developmental, Psychosocial and Learning Problems	15	3.7	(2.1)	6.9	(10.0)	0.2	(0.6)	0.0	(0.0)
Drugs and Alcohol	3	3.1	(2.2)	19.7	(17.4)	0.0	(0.0)	0.0	(0.0)
Ear, Nose and Throat Disorders	7	2.1	(1.3)	16.8	(19.7)	0.0	(0.0)	0.0	(0.0)
Effective Practice and Organisation of Care	5	3.7	(1.9)	18.6	(27.1)	0.0	(0.0)	0.0	(0.0)
Epilepsy	6	2.5	(1.8)	13.8	(15.1)	0.5	(0.8)	0.0	(0.0)
Eyes and Vision	19	2.0	(1.0)	7.6	(10.4)	0.0	(0.0)	0.1	(0.5)
Gynaecological Cancer	7	1.0	(1.6)	13.0	(11.5)	0.4	(0.8)	0.0	(0.0)
Heart	4	3.2	(2.2)	7.3	(3.1)	0.0	(0.0)	0.0	(0.0)
Hepato-Biliary	10	2.4	(0.9)	4.7	(10.2)	0.0	(0.0)	0.0	(0.0)
HIV/AIDS	6	2.5	(1.3)	12.5	(7.3)	0.0	(0.0)	0.0	(0.0)
Hypertension	2	1.1	(0.3)	20.0	(21.2)	0.0	(0.0)	0.5	(0.7)
Incontinence	2	4.1	(0.9)	3.0	(2.8)	0.0	(0.0)	0.0	(0.0)
Infectious Diseases	4	2.9	(1.7)	9.5	(11.0)	0.3	(0.5)	0.3	(0.5)
Inflammatory Bowel Disease and Functional Bowel Disorders	2	5.7	(5.3)	2.0	(1.4)	0.0	(0.0)	0.0	(0.0)
Injuries	14	2.9	(2.0)	8.3	(6.8)	0.1	(0.5)	0.0	(0.0)
Lung Cancer	3	1.8	(1.1)	15.0	(14.5)	0.0	(0.0)	0.0	(0.0)
Menstrual Disorders and Subfertility	8	2.1	(0.9)	21.8	(42.5)	0.0	(0.0)	1.1	(3.2)
Metabolic and Endocrine Disorders	5	3.0	(1.3)	12.6	(11.3)	0.8	(1.8)	0.0	(0.0)
Movement Disorders	6	6.4	(4.2)	7.5	(8.0)	0.2	(0.4)	0.0	(0.0)
Multiple Sclerosis	2	1.5	(1.5)	3.5	(4.9)	0.5	(0.7)	0.0	(0.0)
Musculoskeletal	3	2.3	(1.5)	9.0	(7.0)	0.0	(0.0)	0.0	(0.0)
Neonatal	23	3.9	(2.6)	5.4	(4.3)	0.0	(0.0)	0.0	(0.0)
Neuromuscular Disease	14	2.2	(1.1)	13.9	(15.7)	0.2	(0.4)	0.0	(0.0)
Oral Health	18	2.1	(1.7)	6.4	(10.3)	0.3	(0.8)	0.1	(0.2)
Pain, Palliative and Supportive Care	16	2.8	(2.7)	11.4	(11.1)	0.0	(0.0)	0.2	(0.5)
Peripheral Vascular Disease	5	2.5	(1.2)	10.8	(12.2)	0.2	(0.4)	0.0	(0.0)
Pregnancy and Childbirth	35	2.6	(1.9)	5.2	(11.8)	0.1	(0.4)	0.0	(0.0)
Renal	3	1.6	(1.8)	14.0	(15.1)	2.0	(2.0)	0.0	(0.0)
Schizophrenia	18	5.6	(3.0)	16.0	(11.7)	0.2	(0.5)	0.3	(1.2)
Skin	3	7.3	(5.4)	9.3	(15.3)	0.0	(0.0)	0.0	(0.0)
Stroke	7	4.8	(2.8)	4.3	(6.3)	0.4	(0.5)	0.0	(0.0)
Tobacco Addiction	3	2.1	(0.8)	11.7	(18.5)	0.3	(0.6)	0.0	(0.0)
Upper Gastrointestinal and Pancreatic Diseases	3	1.1	(0.5)	39.0	(56.3)	0.0	(0.0)	1.7	(2.9)
Wounds	5	2.2	(1.5)	14.0	(17.0)	0.2	(0.4)	1.0	(2.2)
**All Review Groups**	**376**	**2.8**	**(2.2)**	**9.6**	**(14.5)**	**0.1**	**(0.5)**	**0.1**	**(0.7)**

The number of excluded studies reported within empty reviews ranged from 0 to 124, with an average of 9.6 (*SD* = 14.5). Ninety-five (25.3%) empty reviews did not report excluded studies. There appears to be some variability in numbers of excluded studies reported within empty reviews across Cochrane Review Groups, ranging from a mean of 2.0 (*SD* = 1.4) in the Inflammatory Bowel Disease and Functional Bowel Disorders Group to an average of 39.0 (*SD* = 56.3) in the Upper Gastrointestinal and Pancreatic Diseases Group. There were only three empty reviews in the latter Review Group, one of which listed 104 excluded studies and an outlier.

The number of on-going studies reported in empty reviews ranged from 0 to 4, with an average of 0.1 listed per review (*SD* = 0.5). Thirty-seven reviews (9.8%) listed at least one on-going study. Only 19 of the 45 Cochrane Review Groups sustaining empty reviews reported on-going studies in these reviews, with the Renal Group reporting the highest average number of on-going studies per review (*M* = 2.0; *SD* = 2.0).

The number of studies awaiting assessment reported by empty reviews ranged from 0 to 9, with an average of 0.1 listed per review (*SD* = 0.7). Only 15 empty reviews (4.0%) and 11 of the 45 Cochrane Review Groups hosting empty reviews reported studies awaiting assessment, with the Upper Gastrointestinal and Pancreatic Diseases Group reporting an average of 1.9 such studies per review (*SD* = 2.9).

Finally, 88 (23.4%) empty reviews did not report any excluded studies, studies awaiting assessment, or on-going studies. More than half of reviews not reporting any studies found in their search were hosted by six Cochrane Review Groups: Cystic Fibrosis and Genetic Disorders, Developmental, Psychosocial and Learning Problems, Eyes and Vision, Hepato-Biliary, Neuromuscular Diseases, and Pregnancy and Childbirth Groups.

## Discussion

### Summary of Results

This study examined empty systematic reviews in *The CDSR*, the world's largest library of systematic reviews. Almost 9% of *The CDSR* consisted of empty reviews, with nearly 85% of Cochrane Review Groups hosting at least one empty review. Nearly half of empty reviews had been published since 2008, whereas 28 (7.4%) reviews were 10 years or older. Nearly half of empty reviews had been updated within the last 2 years, but 15% of empty reviews had not been updated within the past 5 years. The number of excluded studies listed within empty reviews was highly variable (*M* = 9.7, *SD* = 14.5) although more than 25% of these reviews did not report any excluded studies. Further, only 10% of empty reviews reported on-going studies and fewer than 5% of empty reviews reported studies awaiting assessment. Finally, nearly a quarter of empty reviews did not reference any excluded studies, on-going studies or studies awaiting assessment. We found considerable variation across Cochrane Review Groups in terms of numbers and proportions of empty reviews hosted, and at least some variation in time since update, number of excluded studies reported, number of ongoing studies reported, and number of studies awaiting assessment reported.

### Implication of Results

Nearly 9% of systematic reviews published in The CDSR on August 15, 2010 had no studies meeting inclusion criteria. Some of these reviews had remained without included studies for more than 10 years. Findings related to age of reviews provide at most a rough estimate of how long empty reviews persist, because studies meeting inclusion criteria may be found on update. The method to identify empty reviews only accounted for those which were empty on August 15, 2010. Reliable information about how long empty reviews remain empty will require examination of all Cochrane reviews to identify previously empty reviews in future research.

The majority of empty reviews (84.8%) were last assessed as up-to-date within the past 5 years, and nearly half (48.7%) had been updated within the past 2 years. Kristiansen reported that although there were about 3200 published Cochrane reviews as of November 2001, there were only 100–200 updates per year [7]. Clarke and colleagues disputed Kristiansen's numbers but admitted that the Collaboration had room to improve its updating practices [8]. To the extent that update rates across *The CDSR* are similar to those found by Kristiansen, empty reviews would appear to be updated at least as often as reviews with included studies.

Some of the observed differences in reporting of empty reviews across Cochrane Review Groups may relate to differing levels of urgency of questions addressed within topic areas, the ways in which questions are posed or the stringency of inclusion criteria considered across Cochrane Review Groups. On the other hand, observed differences in proportions of empty reviews across Cochrane Review Groups might suggest differing editorial practices and informal policies related to the acceptability of empty reviews.

It may be that many empty reviews result from the problem outlined by Cooper, that is to say that the authors of these reviews are attempting to bring together evidence in the topic area that is immature and, arguably, not currently suitable for review [4]. On the other hand, it may be that the priorities of health care decision-makers and those of researchers do not fit together very well. In either case, the absence of evidence in the empty review might help stimulate appropriate research, resulting in eventual updating of empty reviews with eligible studies.

Explanations for the existence and persistence of these empty reviews remain unknown and warrant further analysis, perhaps through continued monitoring of presently identified empty reviews over time or through detailed qualitative analysis of types of questions posed by the reviews, their inclusion criteria, or the types of conclusions derived from the absence of included studies. Examination of the breadth of questions addressed and inclusion and exclusion criteria may be helpful to understanding the genesis of empty reviews. In addition, examination of quantity and quality of excluded studies reported, reasons for their exclusion, their incorporation in the discussion of implications for practice, and any caveats related to the use of evidence from inferior studies may assist in identifying optimal, and less than optimal, strategies for reporting empty reviews. As the next step in our investigation of empty reviews in *The CDSR*, we have begun to examine the extent to which these reviews contain specific recommendations for practice and whether these recommendations reflect the absence of included studies in the review or if they incorporate information from other sources. We anticipate being able to report the findings of our continued explorations as subsequent steps are completed.

### Limitations

Although very unlikely, it is possible that empty reviews were missed in our search and that the number of these reviews is thus slightly underrepresented. Analysis of the age of empty reviews will require detailed examination of the history of all Cochrane systematic reviews and remains for future research. In addition, the analysis of update status of empty reviews may reflect the update status of all systematic reviews in *The CDSR*, and not reveal anything unique to empty reviews. Further, our assumption that reporting of excluded studies, ongoing studies, or studies awaiting assessment in empty reviews suggests greater promise of eventual update with includable studies may not be valid, although this point warrants further exploration. In particular, studies awaiting assessment may never be assessed, and ongoing studies, when finished, may not meet inclusion criteria.

Perhaps the most important limitation of our study was the examination of empty reviews at only one point in time. While this snapshot of *The CDSR* permitted analysis of incidence and prevalence of empty reviews across *The CDSR* and within Cochrane Review Groups, it did not allow examination of reviews which were previously empty but which have since been updated with eligible studies. Further, this snapshot examination of empty reviews did not permit detection of differences in patterns of updating across Cochrane Review Groups. Thus, our inability to observe the life cycle of empty reviews limits our ability to speculate about the reasons for inconsistencies in incidence and prevalence, updating patterns, or reporting differences across groups.

### Conclusions

The stated purpose of Cochrane reviews is to help healthcare providers, consumers, researchers, and policy makers “make well-informed decisions about health care… by providing a reliable synthesis of *the available* evidence on a given topic… considering *all the evidence* on the effect of an intervention” [2]. Review authors are currently not given specific guidance on how to report empty reviews, and it is clear that such guidance may be necessary. Work toward development of guidance for reporting empty reviews might benefit from a consensus meeting of systematic review contributors and other stakeholders, informed in part by this study and future explorations. Provisional guidance emerging from such a meeting would then require an iterative revision process following methodology outlined by Moher [9]. Guidance for reporting empty reviews might include clear instructions for the incorporation of potentially important information not always considered in systematic reviews of interventions, as well as specific direction on whether to discuss and how to present the findings of non-included studies. Such guidance may even have implications for the reporting of reviews which have included studies.
